# Development of diagnostic tools for IBDV detection using plants as bioreactors

**DOI:** 10.1186/s13568-020-01029-z

**Published:** 2020-05-20

**Authors:** Evangelina Gómez, María Florencia Cassani, María Soledad Lucero, Viviana Parreño, Silvina Chimeno Zoth, Analía Berinstein

**Affiliations:** 1 Instituto de Agrobiotecnología y Biología Molecular (IABIMO), Instituto Nacional de Tecnología Agropecuaria (INTA), Consejo Nacional de Investigaciones Científicas y Técnicas (CONICET), De los Reseros y Nicolás Repetto s/n, 1686 Hurlingham, Buenos Aires Argentina; 2 INCUINTA & Instituto de Virología e Innovaciones Tecnológicas (IVIT), Instituto Nacional de Tecnología Agropecuaria (INTA), Consejo Nacional de Investigaciones Científicas y Técnicas (CONICET), De los Reseros y Nicolás Repetto s/n, 1686 Hurlingham, Buenos Aires Argentina; 3 Present Address: Universidad Nacional de Luján, Ruta 5 y Avenida Constitución, 6700 Luján, Buenos Aires Argentina

**Keywords:** IBDV, In-house ELISA, Molecular farming, Recombinant SVP

## Abstract

Infectious bursal disease virus (IBDV) is the etiological agent of an immunosuppressive and highly contagious disease that affects young birds, thus causing important economic losses in the poultry industry. Multimeric particles with different architectures based on the capsid protein VP2 have been widely produced for different purposes. We hereby show the production and easy recovery of IBDV subviral particles (SVP) from transiently transformed *Nicotiana benthamiana*. The SVP, which were observed by electronic microscopy, proved to be antigenically and immunogenically similar to the virion. Indeed, anti-IBDV antibodies from samples of infected birds recognized these SVP and, when injected intramuscularly, these subviral particles also evoked a humoral immune response in chickens. We developed an in-house ELISA using SVP as coating reagent that demonstrated to be highly accurate and in good agreement with a commercial ELISA. This study demonstrates that the recombinant antigen generated and the technology used to produce it are suitable for developing a diagnostic tool against Infectious bursal disease.

## Key points

- IBDV-SVP are easily recovered from transiently transformed *Nicotiana benthamiana.*
- IBDV-SVP maintain the conformational epitopes of the virion.
- Plant-produced SVP is a reliable antigen to develop a highly accurate IBDV ELISA.
- *Nicotiana benthamiana* is a suitable platform to produce diagnostic reagents.

## Introduction

Infectious bursal disease virus (IBDV), a non-enveloped virus within the *Birnaviridae* family, is the etiological agent of an immunosuppressive disease that affects young chickens. The viral genome is composed of two segments of dsRNA, called segment A and B. Segment A is expressed as a polyprotein precursor that undergoes self-cleavage to release pVP2 (the precursor capsid protein of 512 residues), VP3 (the scaffolding protein) and VP4 (the protease). pVP2 undergoes further processing in which cellular and viral proteases and VP2 itself participate to yield mature VP2 (441 residues) and several C-terminal fragments that remain associated with the capsid (Irigoyen et al. 2012). Segment B encodes for VP1, the RNA-dependent RNA polymerase.

When segment A is expressed in heterologous systems, multimeric particles with different architectures are spontaneously assembled (Martinez-Torrecuadrada et al. 2000a; Caston et al. 2001). The individual expression of VP2-441, or the cleavage processing intermediate VP2-452 (452 residues), leads to the assembly of icosahedral T = 1 subviral particles (SVP) of ~ 23 nm formed by 20 trimers of VP2 (Coulibaly et al. 2005; Garriga et al. 2006; Doong et al. 2007; Taghavian et al. 2013). The hypervariable region of VP2 is contained between the amino acid 204 and 344 and possesses the conformational epitopes that elicit protective immunity and the determinants responsible for the interaction with the host cell (Brandt et al. 2001; Letzel et al. 2007).

Infectious bursal disease (IBD) occurs worldwide and its control depends mainly on accurate vaccination programs. Regarding IBD diagnosis, the World Organization for Animal Health (OIE) recommends the use of Enzyme-Linked Immunosorbent Assay (ELISA) for the detection of immune responses mainly to monitor vaccination programs (OIE 2018). To date, several ELISA kits for IBD are on the market and the most popular kits use the whole virus. However, the precursor of VP2 and even the neutralizing epitopes expressed in heterologous systems also perform adequately to detect anti IBDV antibodies (Martinez-Torrecuadrada et al. 2000b; Sahithi et al. 2019).

Plants constitute a competitive platform for the expression of simple and complex molecules and are suitable for the production of virus-like particles (VLP) for human and veterinary vaccines (Schillberg et al. 2019; Rage et al. 2020). VLP are non-infectious particles made of viral proteins that mimic virus structure while retaining the wild type epitopes. They can display and be carriers of auto or heterologous epitopes and, thus, are excellent candidates as diagnostic reagents. However, few studies have documented the use of plants to produce diagnostic reagents for the veterinary field and there are currently no plant-derived products on the market.

The objective of this study was to evaluate plants as possible hosts for the production of highly conformational antigens of IBDV that can be used as a high-quality reagent in a diagnostic tool for IBDV. The characterization included antigenic and immunological assays and the subsequent development of an indirect ELISA. Although SVP have been produced in bacteria, yeast and insect cells (Martinez-Torrecuadrada et al. 2000b; Rogel et al. 2003; Dey et al. 2009; Wang et al. 2016), the production of IBDV-SVP in plants has remained, to the best of our knowledge, unexplored.

## Materials and methods

### Animals

Embryonated eggs laid by specific-pathogen-free White Leghorn hens were purchased from the Instituto Rosenbusch S.A. (CABA, Argentina) and hatched in an automatic incubator (Yonar, CABA, Argentina). Chickens were kept in individual cages with food and water ad libitum. All procedures were performed in agreement with institutional guidelines and approved by the Institutional Animal Care and Use Committee (C.I.C.U.A.E.—CICVyA—INTA, Approval no. 66/2015).

### Construction of plasmids

The original coding region of the tested VP2 antigen (1323 bp, 441 amino acids) was amplified from the VP2 gene previously cloned (GenBank accession JF965438.1, Zanetti et al. 2012). For this PCR we used the forward 5′ **ACCGGT**ATGACAAACCTGCAA 3′ and reverse 5′**CTCGAG**TTATGCTCCTGCAATCT3′ primers with *Age*I and *Xho*I restriction sites (bold letters), respectively, which amplified from position 1 to 1323 bp of the cloned VP2 gene. The amplicon was inserted into the pEAQ-HT vector (Sainsbury et al. 2009). The vectors obtained are named pEAQ-VP2 and pEAQ-GFP all through the text. The plasmids pEAQ-VP2 and pEAQ-GFP (used as control) were introduced by electroporation into the GV3101 strain of *Agrobacterium tumefaciens*. Dr. Lomonossoff of the John Innes Centre (Norwich, UK) kindly provided pEAQ-HT and pEAQ-HT-GFP vectors.

### Agroinfiltration procedure

The recombinant bacteria were cultured in Luria–Bertani medium containing 100 µg/mL Kanamycin, 100 µg/mL Rifampicin and 50 µg/mL Gentamicin for 16 h at 28 °C and then subcultured for 16 h under the same conditions. The cultures were pelleted and the obtained bacteria were resuspended in an infiltration solution [10 mM MES (morpholinoethanesulfonic acid), pH 5.5; 10 mM MgSO_4_ and 100 µM acetosyringone] to an OD_600_ of 0.8–1. Infiltration of 5 to 6-week-old greenhouse-grown *Nicotiana benthamiana* leaves was performed using a needleless syringe. Leaves were harvested 5 days later (Lucero et al. 2019).

### Preparation of SVP

SVP preparation consisted of an adapted protocol based on double sucrose cushions (Peyret 2015). Total proteins were extracted from 25 g of infiltrated leaves in a blender with 3 volumes of chilled buffer containing phosphate-buffered saline (PBS) pH 7.3, complete EDTA-free protease inhibition cocktail tablets (ROCHE, cat No. 04 693 132 001) and 0.04% antifoam O-30 (Sigma Aldrich, St. Louis Missouri, USA). The extracts were filtered through gauze to clear the sample of gross green material. Clarification was performed at 9000×*g* for 15 min at 4 °C. The supernatant was filtered again through a 0.45 µm membrane in a filter device and loaded above 25% (2 mL) and 70% (0.5 mL) w/v layers of sucrose. Finally, ultracentrifugation was performed at 39,000 rpm in a SW41Ti rotor (Beckman) at 4 °C for 2:40 h.

The interface and the bottom fractions were pooled and dialyzed against PBS (pH 7.4) in a cold room, using dialysis tubes with a MWCO of 12–14,000. Dialysis lasted 39 h and consisted of two buffer changes at 16 and 23 h. SVP were kept at − 70 °C until use. Once thawed, all samples were clarified by centrifugation for 2 min at 10,000*g*.

The negative control sample was obtained by infiltrating leaves with agrobacteria harboring pEAQ-GFP. This control underwent the same protocol described above.

### Detection and quantification of protein forming SVP

The amount of VP2 present in SVP was estimated against a bovine serum albumin (BSA) calibration curve. Briefly, 12 µL of two-fold serial dilutions of BSA (from 250 to 7.8 µg/mL) were loaded and resolved in a 12% SDS-PAGE along with 12 µL of the sample of interest. All samples were analyzed in triplicate. The gel was stained with Coomassie Blue reagent for 40 min and the resolved bands were analyzed with Gel-Pro Analyzer software v3.1. Western blot was performed using rabbit anti-VP2 antibody diluted 1:500 in PBS-0.05% Tween 20 (Gómez et al. 2013). The positive control used for Western blot was a crude extract of plants expressing VP2.

### TEM

Samples from the obtained dialysates were loaded onto a copper grid and stained with 2% phosphotungstic acid. The grids were observed under an electronic microscope (CM 200, PHILIPS) at 160 kV. Image acquisition was performed with OLYMPUS iTEM software.

### Stability of plant-derived SVP

Aliquots of SVP were subjected to different temperature conditions: 4 °C, 37 °C and room temperature for 1, 2, 7, 30, or 60 days and then analyzed by ELISA. Stability at 37 °C was monitored for 30 days.

### Immunological characterization

Five 19-day old chickens were vaccinated in a prime/boost scheme with 60 µg of SVP or the control sample, by intramuscular route (IM) in the leg muscle. Both antigens were formulated in Freund’s Complete and Incomplete Adjuvants in the prime and boost inoculations, respectively. Chickens were vaccinated at 0 and 23 days and bled by the wing vein at 0, 14, 23, 30, 43 and 51 days post-inoculation.

Serum samples of the vaccinated chickens were evaluated for the presence of specific antibodies against IBDV with the commercial IDEXX IBD Ab Test (IDEXX Laboratories, Inc., USA). Titers above 396 were considered positive as stated in the manufacturer’s instructions.

### Optimal working dilutions of recombinant antigen, primary, and secondary antibodies

First, the optimal concentration of plant-derived SVP was determined by titration with positive and negative sera for IBDV obtained from vaccinated and healthy chickens from our animal facility. Briefly, 96-well plates (Maxisorp Nunc) were coated with two-fold serial dilutions of SVP in 0.1 M bicarbonate buffer, pH 9.6, overnight at 4 °C. The plate was blocked with 5% skim milk in PBST-ENS (0.05% Tween 20, 5% equine normal serum) and subsequently incubated with anti-IBDV and negative serum diluted 1:100 as primary antibodies and 1:3000 goat anti-chicken IgG antibody coupled to horseradish peroxidase (Bethyl Laboratories Inc cat. # A30-106P). The detection step was performed with H_2_O_2_/ABTS [2,2′-azinobis (3-ethylbenzthiazoline-6-sulfonic acid) diammonium salt] substrate/chromogen system in citric acid buffer, pH 5. Reading was carried out at 405 nm after 30 min of incubation or once the anti-IBDV serum of each plate (positive control) reached the admission rank of absorbance previously determined (Mean OD value = 0.84–1.07).

The best combination between primary and conjugated antibodies was determined performing a checkerboard titration. This titration consisted of testing 1:50, 1:100, 1:200, 1:300, 1:400 and 1:500 dilutions of the anti-IBDV antibody and 1:3000, 1:4000, 1:5000 and 1:6000 dilutions of the anti-chicken conjugated to peroxidase.

### Cut-off value and accuracy measures from ROC analysis

A total of 432 reference sera of chickens classified as true negatives (192 samples) and true positives (240 samples) for IBDV using the commercial IDEXX IBD Ab Test (IDEXX Laboratories, Inc.) were used for ROC analysis of the in-house ELISA-SVP. True positive sera belonged to animals naturally infected or vaccinated from our animal facility or commercial farms. Negative sera came from animals used in past experiments in our lab. The method based on the sensitivity–specificity (Se–Sp) equality criterion was used to compute the optimal cut-off point. The concordance between methods to properly classify the samples as positive or negative was estimated by the calculation of the Weighted Cohen’s kappa coefficient (k test) using a panel of 144 new sera. Values of k test from 0.41 to 0.60 indicate moderate agreement; values from 0.61 to 0.80, substantial agreement, and values from 0.81 to 0.99, almost perfect agreement (Viera and Garrett 2005). The analyses were done using R 3.4.1 (R Core Team 2017), the OptimalCutpoints, and the DescTool packages (López Ratón et al. 2014; Signorell 2019).

## Results

### Production and preparation of SVP

**Fig. 1:**
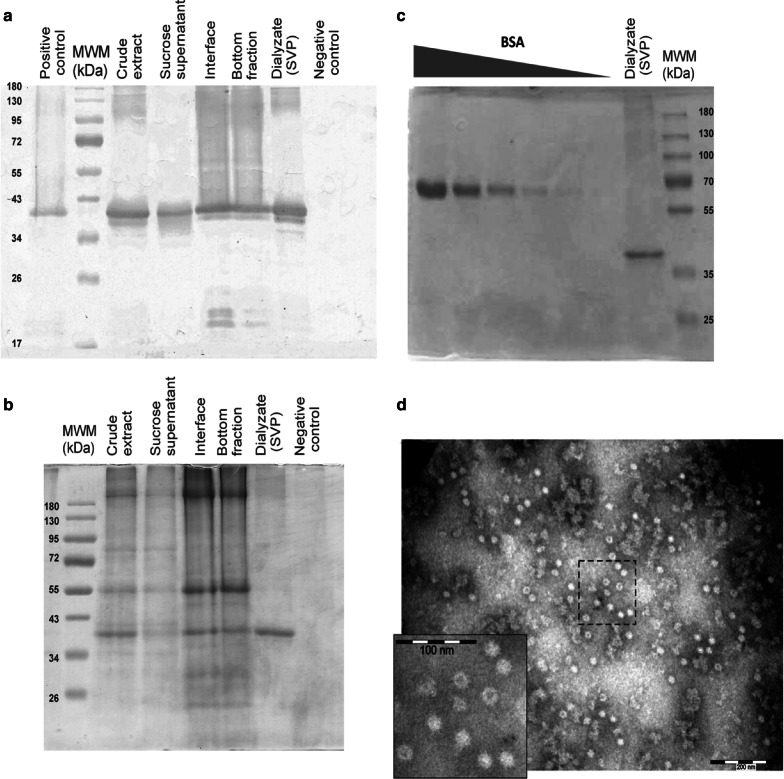
Expression, semipurification, and quantification of SVP. Aliquots of the subsequent steps of the purification of the samples were loaded into 12% SDS-PAGE for **a** immunoblotting and **b** Coomassie blue staining. VP2 were identified using a rabbit anti-VP2 antibody diluted 1:500. The negative control consisted of leaves infiltrated with GFP subjected to the same purification process (see “Materials and methods”). **c** Standard curve of BSA—250, 125, 62.5, 31.25, 15.6 and 7.8 µg/mL—and VP2 were stained with Coomassie blue reagent and the intensity of the bands were analyzed with Gel-Pro Analyzer software v3.1. **d** Transmission electron micrographs of SVP. The dialysate was subjected to negative staining with 2% phosphotungstic acid and observed under an electronic microscope (CM 200, PHILIPS). Scale bar of large pictures = 200 nm

To achieve the production of SVP in *Nicotiana benthamiana*, we used pEAQ-HT vectors known to give high levels of expression or accumulation of recombinant protein in plants, and a simple method of purification consisting in ultracentrifugation followed by dialysis. All steps of the purification procedure [crude extract, sucrose supernatant, interface, bottom fraction (70% sucrose fraction) and dialysate] were analyzed by SDS-PAGE and immunoblotting using an anti VP2 antibody to visualize the recombinant protein. As expected, all samples presented a specific band of the expected molecular weight, which would correspond to the monomer of VP2 (~ 42 kDa; Fig. 1a), except for the negative control.

It is worth mentioning that dialysis and clarification steps helped us to eliminate most contaminants from the pooled sample (Fig. 1b), an unexpected but desirable effect. The method applied here to obtain the particles allowed the retrieval of ~ 7 mL of sample and a recovery of 56 µg VP2/mg of fresh tissue according to the BSA curve (Fig. 1c).

Furthermore, particles of spherical shape and homogeneous size with a diameter of approximately 19.8 ± 1.76 nm were present in the dialysate as evidenced by the electronic microscopy (Fig. 1d).

### Immunological properties of the SVP

**Fig. 2:**
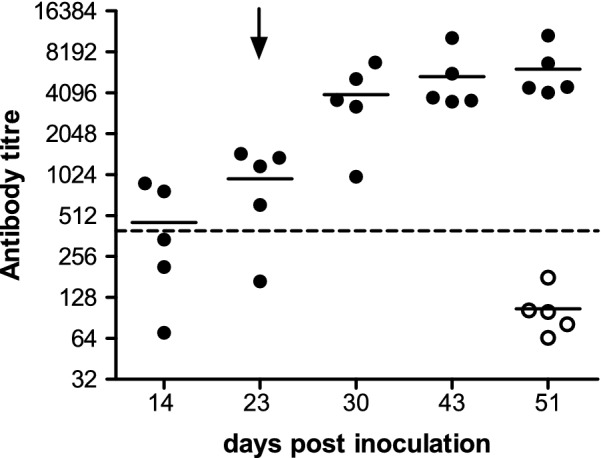
Humoral immune response elicited by SVP. Nineteen-day-old chickens were immunized with SVP (closed circles) or control sample (open circles) on days 0 and 23 and bled at 0, 14, 23, 30, 43, and 51 days post-inoculation. Sera were evaluated for the presence of specific antibodies against IBDV with a commercial kit (IDEXX Laboratories, Inc., USA). Titres above 396 were considered positive as indicated by the manufacturer’s instructions (dashed line). Black lines indicate the mean of the antibody titre. Arrow indicates boost vaccination

In order to study the immunogenicity of the particles, we conducted a vaccination experiment (see “Materials and methods”). Animals vaccinated with SVP developed a humoral response as soon as 14 dpi, which became higher after the boost, as evidenced by the increase of antibody titers throughout the experiment (Fig. 2). As expected, chickens vaccinated with the control sample showed no anti-IBDV antibodies.

The results indicate that the particles maintain the conformational epitopes present in the virion as they can elicit anti-IBDV antibodies.

### ELISA-SVP

**Fig. 3:**
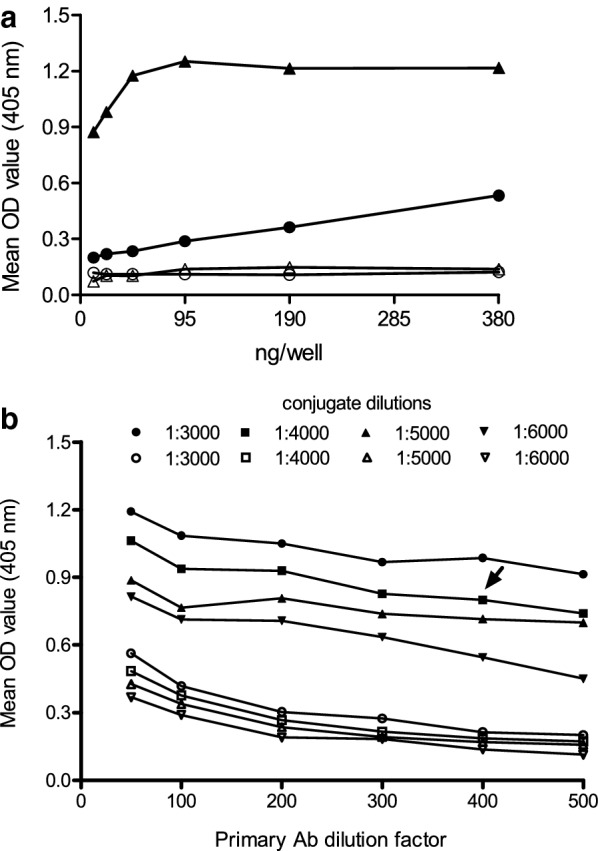
Titration of the ELISA-SVP reagents. **a** Antigen titration. Serial two-fold dilutions of boiled (circles) and non-boiled SVP (triangles) were titrated using 1:100 anti-IBDV and negative serum (open symbols). Anti-chicken antibody conjugated to peroxidase was used in dilution 1:3000. SVP were boiled for 3 min and then assayed. **b** Checkerboard titration between primary and conjugate antibodies. Polyclonal sera anti-IBDV diluted 1:50 to 1:500 and anti-chicken HRP diluted 1:3000 to 1:6000 were assayed using SVP (curves with closed symbols) or control sample (curves with open symbols). The arrow indicates the working dilutions of choice

To determine whether plants can be a suitable platform to produce diagnostic reagents for IBDV, we established the conditions of an indirect ELISA using SVP as antigen, and then compared the results of the ELISA with those of a commercial test. The analysis showed that 95 ng per well of the SVP (1.9 µg/mL, Fig. 3a) along with 1:400 dilution for the anti-IBDV and 1:4000 dilution for the conjugated antibodies (Fig. 3b) turned out to be the optimal dilutions to give maximum signal, minimum background, and a cost-effective assay. The signals of the negative control serum as well as the blank of reaction were very low, as expected.

In addition, boiling SVP before coating the plate produced a drastic decrease in the absorbance (Fig. 3a). This finding suggests that the conformational epitopes of the SVP were seriously damaged by this procedure. This result gives more evidence of the presence of conformational epitopes and is consistent with the fact that SVP triggers an anti-IBDV antibody response (Fig. 2).

**Fig. 4:**
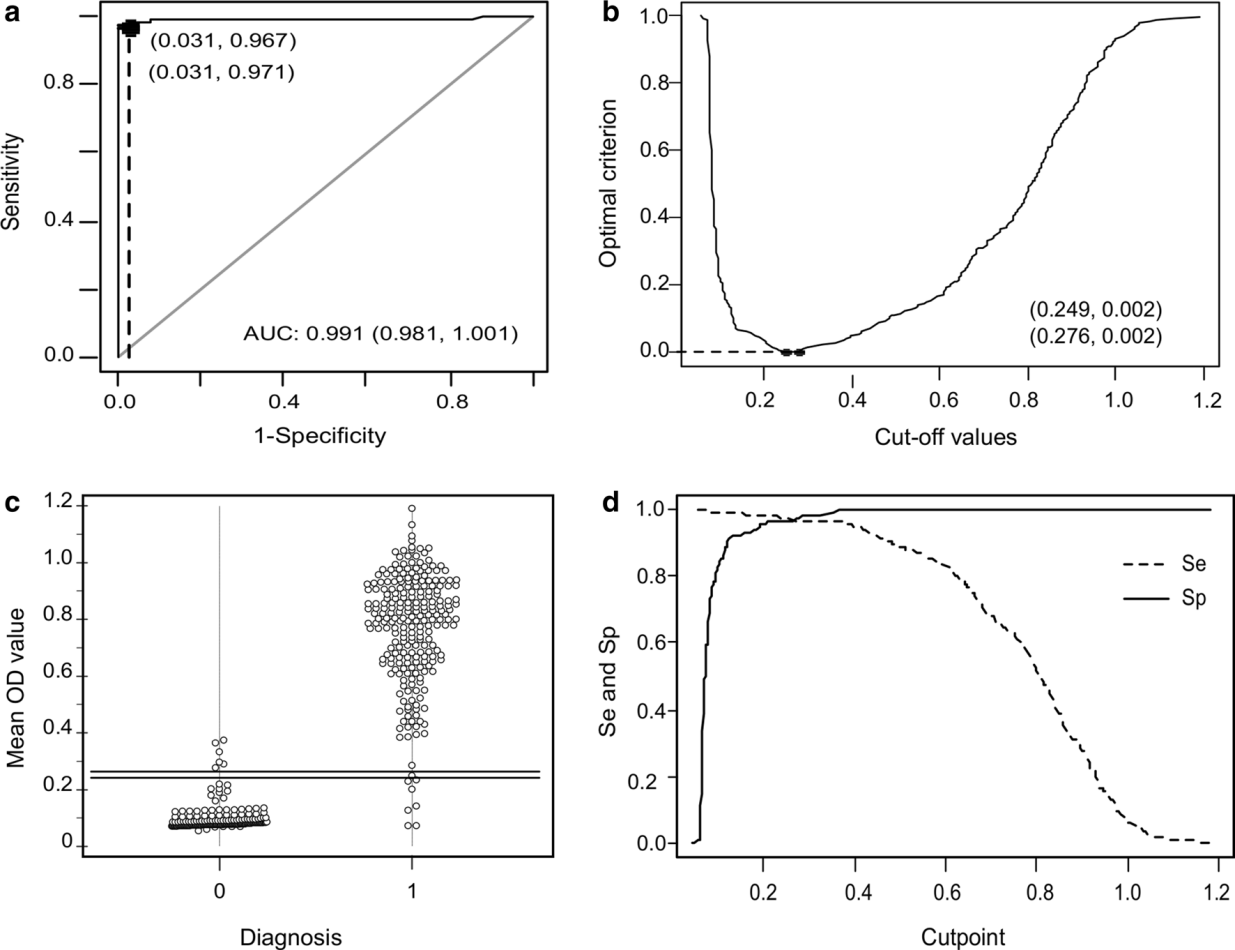
Graphical analysis of the cut off value, Se and Sp for the ELISA-SVP. **a** ROC curve. Numbers in brackets in the upper left corner corresponds to 1 minus Sp and Se values for the resulting cutoffs. **b** Cut off values obtained using Se = Sp criterion. The optimal criterion refers to the Se–Sp difference at the optimal cutoff. **c** Dot diagram for the 432 reference samples, 240 positives (code = 1), and 192 negatives (code = 0) analyzed with the in-house ELISA-SVP. The lines represent the cut off values (0.249 and 0.276 OD values). **d** Sensitivity and specificity plots according to cutpoints

Once the ELISA-SVP conditions were defined, we analyzed 432 reference sera to calculate the accuracy measures of the ELISA-SVP (Table 1) taking the commercial ELISA as reference and obtained the cut-off values (Fig. 4) suggested by ROC analysis using the Se–Sp equality criterion. The area under the ROC curve (AUC) results from plotting the coordinates (Se; 1 − Sp) for all possible cut-off points. The AUC takes values ranging from 0.5 (uninformative test) to 1 (perfect test). Here, the AUC and confidence interval obtained was 0.991 (0.981–1.001); which implies that the ELISA-SVP displays almost perfect ability to discriminate negative from positive sera samples. Two cut-off values (0.249 and 0.276) accomplished the condition of Se equal to Sp. For both cut-off values, most of the samples (97%) correctly classified as positive. Likewise, the same percentage correctly classified as negative. The cut-off value of 0.276 resulted in 8 false negatives, whereas the cut-off value of 0.249 gave 7 false negatives.

**Table 1:** Accuracy measures and ROC analysis for the ELISA-SVP based on Sp and Se equal criterion

	Cut off 0.25		Cut off 0.28		ROC analysis		
	0.95 CI		0.95 CI		AUC	0.95 CI	Correctly classified
Se	0.97	0.94–0.99	0.97	0.93–0.99	0.991	0.981–1.001	99%
Sp	0.97	0.93–0.99	0.97	0.93–0.99			
PPV	0.97	0.94–0.99	0.97	0.94–0.99			
NPV	0.96	0.93–0.99	0.96	0.92–0.99			
FP	6		6				
FN	7		8				

*Se* sensitivity, *Sp* specificity, *PPV* positive predictive value, *NPV* negative predictive value, *FP* false positive, *FN* false negative, *AUC* area under the ROC curve, *CI* confidence interval

The analysis of concordance for both cutoff values, 0.249 and 0.276, gave a Kappa value of 0.95 (0.88–1.02) and 0.92 (0.84–1.01), respectively, with 144 new sera; which implies an almost perfect agreement between them. Therefore, any of the cut-off values could be used in the ELISA-SVP.

### SVP stability

**Fig. 5:**
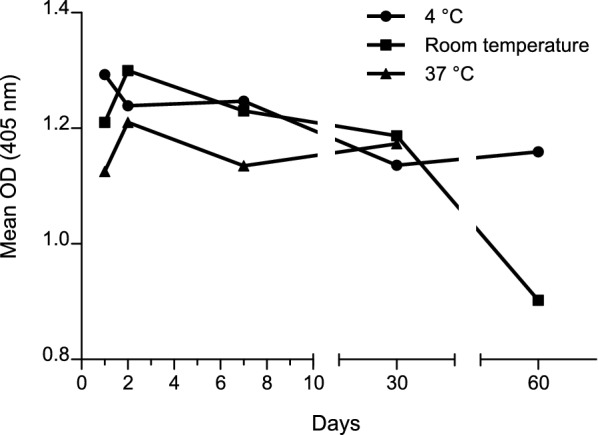
Analysis of IBDV-SVP stability by ELISA. Aliquots of SVP were stored for 1, 2, 7, 30, or 60 days at room temperature (RT), at 4 °C or 37 °C, and then assayed in the ELISA SVP using 1:400 and 1:4000 dilutions of anti-IBDV and anti-chicken HRP antibodies, respectively. Stability at 37 °C was monitored for 30 days

The performance of SVP as an antigen for the ELISA-SVP, subjected to different conditions, was assessed. Aliquots of SVP were incubated at different temperature for 1, 2, 7, 30 or 60 days before tested. Stability at 37 °C was monitored for 30 days. The results indicated that SVP conserved the epitopes for antibody recognition after incubation at 4 °C, 37 °C or room temperature for several days (Fig. 5).

## Discussion

The use of plants as bioreactors to produce valuable molecules has been well documented. Its rationale relies on scalability, cost-effectiveness, and time saving; which turns plant expression systems into an attractive and suitable platform. Expression of complex molecules is also feasible in plants (Scotti and Rybicki 2013; Catrice and Sainsbury 2015; Peyret et al. 2015; Rybicki 2019) and therefore it was our system of choice.

We demonstrated that the recovery of subviral particles from transiently transformed plants is possible through a simple method (sucrose cushion followed by dialysis). To our knowledge, this is the first study showing the production and recovery of IBDV subviral particles from a model plant (*N. benthamiana*). We detected spherical particles of homogeneous size of approximately 19.8 ± 1.76 by electronic microscopy (Fig. 1d). Although smaller than the SVP produced in other expression systems like ~ 23 nm SVP derived from yeast (Taghavian et al. 2013; Wang et al. 2016) and insect cells (Martinez-Torrecuadrada et al. 2003), these plant-derived SVP conserved the antigenic and the immunogenic properties. Difference in size could arise from the measurement tools used among the different studies. A dynamic light scattering analysis may be more appropriate for assessing the exact size of purified plant-derived SVP.

On the other hand, the few steps in the recovery process seem to be sufficient to remove the bulk of the impurities of the sample (Fig. 1a, b). In addition, the in-house ELISA results suggest that further steps of purification would be unnecessary. Indeed, negative sera of chicken were unreactive with the dialysate (SVP) (Fig. 3a) and false positives are extremely low when considering the cut-off values suggested by ROC curves (Table 1).

Several researchers have described the immunogenicity of SVP (Martinez-Torrecuadrada et al. 2003; Taghavian et al. 2013; Wang et al. 2016). Ho and coworkers reported the capsid formation and immunogenic properties of deleted variants in amino acid residues at the N- or C-terminal region of VP2-452 produced in insect cells (Ho et al. 2010). That study showed that both VP2-452H and VP2-441H (VP2 with a His-tag at the c terminal) yielded similar amounts of recombinant proteins. We also performed the expression of VP2-452 in *N. benthamiana* and studied the expression kinetic as explained in materials and methods, but the expression levels turned out to be considerably lower than those of VP2-441 (unpublished results). From our and others’ experiences, the level of recombinant protein is critical to be able to observe SVPs by electronic microscopy. For this reason, we used pEAQ-HT vector and, furthermore, chose mature VP2-441 to obtain the SVP. The results obtained in chickens herein presented agree with those observed by other researchers. Indeed, the results indicate that *N. benthamiana*-derived SVP also conserved the highly conformational epitopes, suggesting that this model plant can be not only a production platform for IBDV antigens but also a vaccine platform for IBD control.

The results from the assessment of the performance of plant-derived SVP as antigen for an ELISA kit revealed that the best temperature to store the SVP is 4 °C, although it is also very stable at room temperature. Rani and Kumar (2015) demonstrated that IBDV subjected to different temperatures for 2 h lost infectivity at 56 °C. This finding suggests that high temperatures might affect the conformation of the capsids (at least the sites responsible for interaction with cell surface receptor), although protein mass remained unaltered even at 72 °C (Rani and Kumar 2015). In another study, purified SVP produced in yeast were resistant to temperatures between 25 and 60 °C for 1 h, as no precipitation or apparent conformational changes occurred (Taghavian et al. 2012). In our study, plant-derived SVP were stable at room temperature and at 37 °C for at least 30 days. Therefore, SVP are not only resistant to high temperatures, as reported before, but also for long periods. In addition, in the previous reports and in our study, the solubility of the protein was completely lost with boiling. Further studies of accelerated stability (at 37 °C) and stability in working conditions (at 4 °C) with a pilot batch made of raw materials of choice, such as plastic and preservatives, should be performed to determine the optimal temperature of storage and expiration date.

Altogether, we demonstrated that SVP conserved the epitopes responsible for the interaction with anti-IBDV antibodies at cold or at room temperature in PBS, which is a very attractive feature for an antigen intended to be used in an ELISA kit.

In a previous study, the researchers of two different groups reported optimal SVP concentrations of 0.5–1.5 µg/well or 1 µg/mL to be used in an in-house ELISA (Martinez-Torrecuadrada et al. 2000b; Dey et al. 2009). Similarly, in this study, the optimal concentration was 95 ng/well, which corresponds to 1.9 µg/mL, of the plant-derived antigen. Therefore, our findings agree with those expected for recombinant SVP.

Finally, we tested its performance in comparison with the ELISA IDEXX, one of the most popular commercial ELISA kits used for the detection of anti-IBDV antibodies. The cut-offs suggested by the ROC curves had a 97% of specificity and a 97% of sensitivity associated and an AUC of 0.99. This means that the ELISA-SVP will correctly classify samples in 99 out of 100 times, thus indicating high accuracy of the tests. Kappa test using either of the obtained cut-offs also indicated a good force of agreement between tests (k > 0.9). It is worth mentioning that a third serological test could be applied to classify the discordant samples.

The use of the recombinant antigen proposed in this study avoids the manipulation, amplification (e.g. in eggs or in cell culture) and purification of an infectious agent and furthermore decreases the costs associated to these processes. In addition, both ELISAs performed well suggesting that the recombinant ELISA-SVP could replace the commonly used test.

We demonstrated that *N. benthamiana* is a suitable platform for producing efficiently assembled IBD SVP that conserve antigenic and immunogenic properties. We also showed that a simple method of semipurification is enough to recover recombinant subviral particles from leaves and that these particles, in turn, can be used as a reliable antigen for an ELISA kit. Therefore, this plant-derived technology has great potential as a platform for producing diagnostic tools for infectious bursal disease.

## Data Availability

All data generated or analyzed during this study are included in this published article.

## Abbreviations

IBDV: Infectious bursal disease virus
SVP: Subviral particles
IBD: Infectious bursal disease
OIE: World Organization for Animal Health
ELISA: Enzyme-Linked Immunosorbent Assay
VLP: Virus-like particles
PBS: Phosphate-buffered saline
BSA: Bovine serum albumin
IM: Intramuscular route
Se: Sensitivity
SP: Specificity
AUC: Area under the ROC curve
